# Direction-Dependent Effects of Combined Static and ELF Magnetic Fields on Cell Proliferation and Superoxide Radical Production

**DOI:** 10.1155/2017/5675086

**Published:** 2017-04-12

**Authors:** Jonne Naarala, Kavindra Kumar Kesari, Ian McClure, Cristina Chavarriaga, Jukka Juutilainen, Carlos F. Martino

**Affiliations:** ^1^Department of Environmental and Biological Sciences, University of Eastern Finland, P.O. Box 1627, 70211 Kuopio, Finland; ^2^Department of Biomedical Engineering, Florida Institute of Technology, Melbourne, FL 32901, USA

## Abstract

Proliferation of human umbilical vein endothelial cells was stimulated by a nearly vertical 60 or 120 *μ*T static magnetic field (MF) in comparison to cells that were shielded against MFs. When the static field was combined with an extremely low frequency (ELF) MF (18 Hz, 30 *μ*T), proliferation was suppressed by a horizontal but not by a vertical ELF field. As these results suggested that the effects of an ELF MF depend on its direction in relation to the static MF, independent experiments were carried out to confirm such dependence using 50 Hz MFs and a different experimental model. Cytosolic superoxide level in rat glioma C6 cells exposed in the presence of a nearly vertical 33 *μ*T static MF was increased by a horizontal 50 Hz, 30 *μ*T MF, but not affected by a vertical 50 Hz MF. The results suggest that a weak ELF MF may interact with the static geomagnetic field in producing biological effects, but the effect depends on the relative directions of the static and ELF MFs.

## 1. Introduction

Extremely low frequency (ELF) magnetic fields (MF) have been classified as “possibly carcinogenic to humans” [1], mainly based on rather consistent epidemiological evidence suggesting an association between power line ELF EMFs and childhood leukemia. The epidemiological associations have been reported at very low magnetic field levels (0.3–0.4 *μ*T), but the causality of these associations is not clear, and there are no generally accepted mechanisms for effects from such weak MFs.

Several animal species are able to detect the geomagnetic field and changes in it for the purposes of orientation and navigation. Such animal magnetoreception is believed to be based on MF effects on radical pair reactions [2] and/or on biogenic magnetite particles [3]. These mechanisms could be sensitive enough to explain adverse health effects of ELF MFs, but it is not clear at present how 0.4 *μ*T alternating MFs could lead to significant biological effects in the presence of the much stronger static MF of the earth.

Burda et al. [4] reported that the alignment of grazing and resting cattle and deer was disrupted by power lines, and the disruption was affected by the relative directions of the static geomagnetic field and the alternating MF from the power lines. Vanderstraeten and Gillis [5] discussed disruption of static MF effects by alternating fields that are either parallel or perpendicular with the static field (causing oscillations in the intensity or direction of the MF). They concluded that such oscillations can be transduced by both radical pair-based and at least certain iron mineral-based mechanisms.

We hypothesize that the basic interaction mechanisms are the same in animal orientation and in cellular effects of MFs, and the in vitro effects of an alternating MF could therefore depend on the relative directions of the alternating and static fields. Data from one laboratory (first series of experiments, described below) seemed to support this hypothesis, so we decided to carry out an independent test in another laboratory with higher number of replicates but simplified study design. The results of both series of experiments are reported in this paper. Both laboratories used their own exposure setups and in vitro experimental models that they had used in previous studies on MFs. Also the frequency of the alternating MFs used in the second series of experiments (50 Hz) differed from that used in the first series of experiments (18 Hz). We did not harmonize these experimental details, as use of different approaches was considered to shed light on the generalizability of the results and to elucidate the mechanism of action. Proliferation of human umbilical vein endothelial cells (HUVEC) was assessed in the first series of experiments. This model was chosen based on previous experiments showing decreased proliferation in cell cultures shielded against MFs in comparison to cells kept in 43–120 *μ*T static MFs [6, 7]. In the second series of experiments, mitochondrial and cytosolic superoxide levels were studied in rat glioma C6 cells. This model was chosen because of previous findings showing increased superoxide levels in this cell line after 24 h exposure to 50 Hz magnetic fields at 10–30 *μ*T [8]. The preliminary experiments with HUVEC cells included testing the effects of a near-zero MF (cells shielded against MFs), as well as those of horizontal or vertical alternating MFs. Also, the experiments included two different static MF strengths (60 *μ*T and 120 *μ*T) in the control group. The further experiments with C6 cells were simplified and focused on just confirming (with a higher number of replicates) different responses to horizontal and vertical alternating MFs in the presence of a (nearly) vertical static MF.

## 2. Materials and Methods 

### 2.1. Magnetic Field Exposure

**Figure 1:**
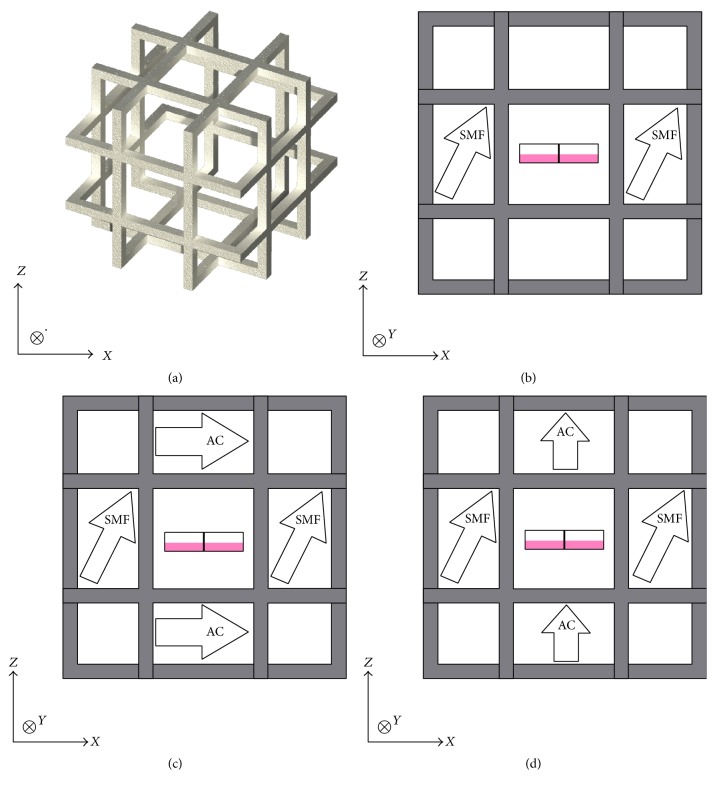
Magnetic field exposure conditions of HUVEC cells. (a) Three-axial coil system for generating the static magnetic field (SMF) and the alternating current (AC) magnetic fields. (b) Side view of the coil system when SMF alone was used. (c) Side view when SMF was combined with a horizontal AC field. (d) Side view when SMF was combined with a vertical AC field. The rectangle in the middle represents side view of the cell culture plate. The sides of the square coils measured 20–25 cm and the coils were separated by 10–12 cm.

The initial background static MF inside the incubators (Binder CB 150, Germany) used for the HUVEC cells varied from 10 to 40 *μ*T, so triaxial single-wound Helmholtz coils were used to establish a uniform static MF and vertical or horizontal ELF MFs (Figure 1). Each coil consisted of 20 turns of 22 gauge copper wire. The side of each square coil measured 20–25 cm and each pair of coils was separated by 10–12 cm. A function generator HP33120A (Hewlett-Packard, Palo Alto, CA) was connected directly to the coils for generation of 18-Hz vertical or horizontal MFs. Near-zero MF conditions were produced by a horizontally rested *μ*-metal cylinder with a radius of 12 cm and length of 30 cm. The cylinder allowed free flow of CO_2_-air mixture but attenuated the static MF to 0.2 *μ*T–2.0 *μ*T over an 8 cm by 12 cm volume in the middle of the cylinder (FW Bell, Rochester, NY). Cells were exposed in 8 by 12 cm 6-well plates (TPP, Germany) centered vertically and horizontally between the coils. Cells were seeded and allowed to rest for 24 h in the ambient geomagnetic field, which was 30–50 *μ*T in the incubator. Magnetic field exposure conditions (Figure 1) were a reference group exposed to a 60 or 120 *μ*Τ static MF at 30° to the vertical axis and three experimental conditions: (a) the same static MF combined with a 18-Hz, 30 *μ*T rms vertical magnetic field, (b) the same static MF combined with 18-Hz, 30 *μ*T rms horizontal magnetic field, and (c) near-zero MF. Static MFs were measured with IDR-310 and low frequency MFs with a IDR-210 gaussmeter (Integrity Design, VT).

**Figure 2:**
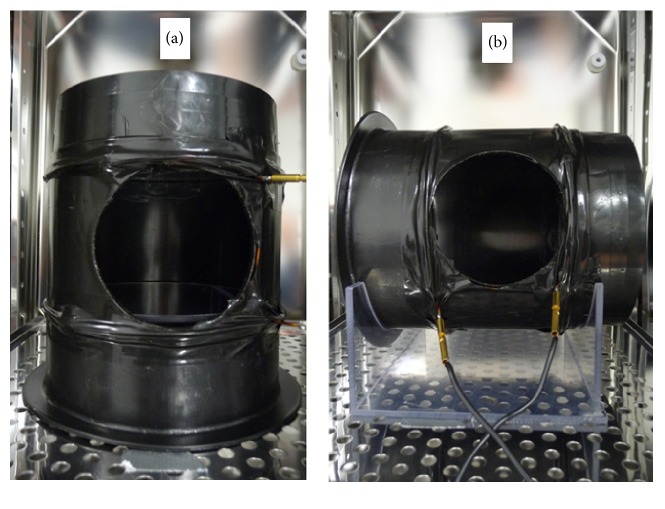
Magnetic field exposure of C6 cells. (a) Helmholtz coils set up to generate a vertical alternating magnetic field. (b) Helmholtz coils set up to generate a horizontal alternating magnetic field perpendicular to the static geomagnetic field. The coils were 21 cm in diameter, and their separation was 10.5 cm.

Circular single-wound Helmholtz coils were used for exposing the C6 cells. Each coil consisted of 5 turns of 2 mm copper wire. The radius of the coils was 10.5 cm, and the distance between the two coils in a Helmholtz pair was 10.5 cm. The coil system can be turned to produce either vertical or horizontal MFs (Figure 2). Two identical coil systems were placed in two identical incubators (HERAcell, Heraeus, Germany) for simultaneous MF and sham exposure, with no current connected to the sham exposure coil. Due to the low resistance of the 5-turn coils, the power dissipated in them is low (7.5 mW when a 30 *μ*T MF is produced). Heating of the cell cultures should therefore be negligible. Temperature measurements with Fluke 52 K/J Thermometer (John Fluke Mfg. Co. Inc., USA) for 24 h confirmed that there was no temperature difference (greater than 0.1°C) between the exposure and sham-exposure systems even when a 300 *μ*T alternating MF was generated. No active adjustment of the static MF was used in this exposure system. The geomagnetic field measured with Hirst GM08 Gaussmeter and Hirst Axial Fluxgate Probe AFG100 (Hirst Magnetic Instruments Ltd., Cornwall, UK) was about 33 *μ*T and almost vertical (inclination 80–85°) in the incubators. For horizontal MF exposure, the direction of the Helmholtz coil system was chosen so that the AC magnetic field oscillated in the magnetic east-west direction and was thus perpendicular to the geomagnetic field. The orientation of the sham-exposure coil was always the same as that of the active coil.

### 2.2. Culture of HUVEC Cells and Proliferation Assay

Human umbilical vein endothelial cells (HUVECs) isolated as previously described [9] (passages 2–4, 2–4 weeks old) were cultured in endothelial growth medium (Promocell, Heidelberg, Germany) supplemented with 10% fetal calf serum (Promocell, Heidelberg, Germany), 0.004 mL/mL endothelial cell growth supplement/heparin, 0.1 ng/mL epidermal growth factor, 1 ng/mL basic fibroblast growth factor, and 1 *μ*g/mL hydrocortisone (EGF, Promocell, Heidelberg, Germany) at 37°C with 5% CO_2_. The cells were cultured in a 75 cm^2^ flask to expand cell number. For the cell counting assay, 6-well culture plates were seeded with 8.0 × 10^4^ cells per well and incubated in 5% CO_2_ at 37°C for one day prior to MF exposure. After exposure for 2 days, the cells in 3 wells were counted 3 times using a cell counter (Casy Model TT, Bielefeld, Germany). Two replicate experiments were performed. The experiments were blinded; that is, the technician performed the measurements without knowledge of the treatments.

### 2.3. Culture of C6 Cells and Superoxide Assays

The rat C6 glioma cell line (acquired from Professor Nikolaus Plesnila, Institute for Stroke and Dementia Research (ISD), LMU Munich Medical School, Munich, Germany) was grown in DMEM containing 1 g/L glucose, 10% FBS, and a mixture of 50 U/mL penicillin/50 mg/mL streptomycin. The cells were maintained at 37°C and 5% CO_2_ in a humidified atmosphere. The cells were detached by 0.02% EDTA (prepared in Ca^2+^- and Mg^2+^-free phosphate-buffered saline), with 0.1% trypsin added. For the superoxide assays, 3 × 10^4^ cells were seeded on 48-well plates (Costar, Corning, NY, USA) 20 h prior to the onset of MF exposure.

As described previously [10], production of cytosolic superoxide was measured by the DHE (dihydroethidium) probe using a final concentration of 10 *μ*M and 485 nm excitation/595 nm emission wavelengths. Mitochondrial superoxide levels were measured by the MitoSOX Red probe (1 *μ*M final concentration) at 492 nm excitation/595 nm emission wavelengths. Immediately after the exposures, the medium was removed from the 48-well plates and the cell cultures were loaded (30 min, +20°C, in dark) with the assay-specific probe in 0.5 mL of phosphate buffer saline (PBS). Thereafter, fluorescence was measured by a multiwell fluorometer (Tecan Infinite F200 Pro, Tecan GmbH, Austria). A blank (no cells) was included in all measurements. Blank values were subtracted from the absolute values. Five samples per exposure group were used, and the experiment was repeated 4 times. Out of 160 values measured by the DHE probe, 7 had to be rejected because of impossible values (negative fluorescence, indicating a possible technical error). These occurred in 2 MF-exposed and 2 sham-exposed samples in the horizontal MF experiments and in 2 MF-exposed and 1 sham-exposed sample in the vertical MF experiments. The experiments were not done in blinded manner, as the superoxide measurements were done using an automated measuring instrument, and the experimenter had no possibility of influencing the results.

### 2.4. Statistical Analysis

Two-way ANOVA was performed by the GraphPad Prism 5.03 software (GraphPad Software Inc., La Jolla, CA, USA), with MF treatment and replicate included as factors in the model. Bonferroni posttests were used to test differences between individual groups.

## 3. Results

**Figure 3:**
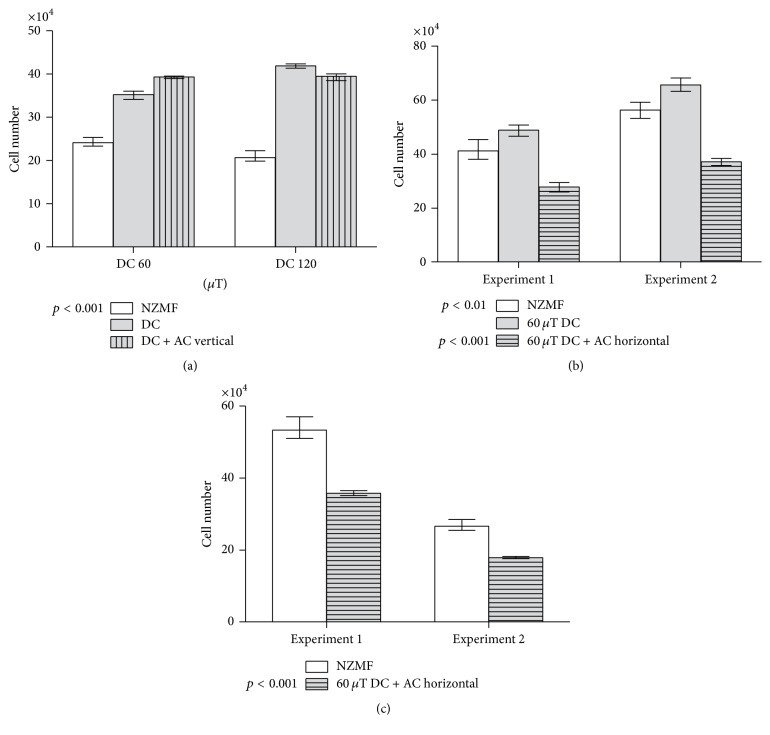
Effects of static and alternating magnetic fields (MF) on proliferation of HUVEC cells: number of cells (±SD) measured after 2 days of growth. (a) Cells grown in near-zero MF (NZMF), nearly vertical static (DC) MF at two magnetic flux densities, or combination of the DC field and a vertically oscillating 18-Hz alternating (AC) MF. (b) Cells grown in NZMF, nearly vertical 60 *μ*T DC MF, or combination of the DC field and a horizontally oscillating 18-Hz AC MF. (c) Further experiment comparing cells grown in NZMF or combination of the DC field and the horizontally oscillating AC field. Significant differences from the DC treatment (in (a) and (b)) or from the NZMF treatment (in c) are shown. The overall difference between the MF treatment groups was in all cases significant at *p* < 0.001.

Consistent with previous findings [6, 7, 11], decreased proliferation of HUVEC cells was found in the near-zero MF in comparison to the reference group exposed to a static MF (Figure 3(a)). Cell proliferation was not consistently affected by a vertical ELF MF (18 Hz, 30 *μ*T) superimposed on the static MF: a suggestive small increase was seen when the static MF was 60 *μ*T, but this was not replicated in the experiment with a 120 *μ*T static field. In cells exposed to the horizontal ELF MF, in contrast, cell proliferation rate was only about half of that of the reference group (Figure 3(b)). Reduced proliferation in the near-zero MF was observed again in these experiments, but the data suggested that the horizontal AC field might inhibit proliferation even more than the near-zero MF. This was confirmed in additional experiments comparing only the near-zero MF versus the horizontal AC field combined with a 60 *μ*T static field (Figure 3(c)).

**Figure 4:**
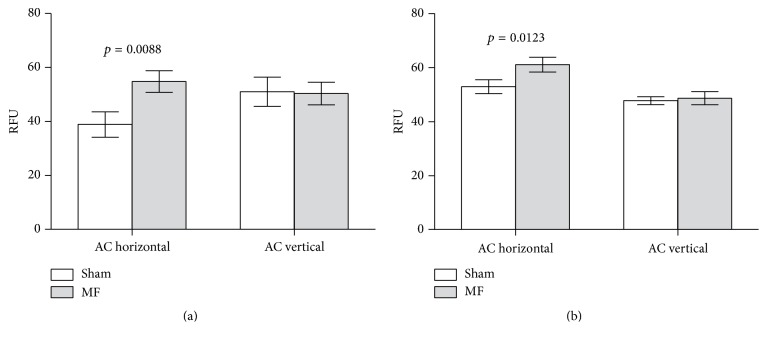
Effects of horizontal or vertical alternating (AC) magnetic fields (MF) on cytosolic superoxide level in C6 rat glioma cells: relative fluorescent units (RFU) in cells exposed for 24 h. (a) Samples assayed 3 h after the end of MF exposure. (b) Samples assayed immediately after the end of MF exposure. *p* values for significant differences between sham and MF treatments are shown.

The difference between the effects of vertical and horizontal ELF magnetic fields was confirmed in C6 cells exposed to 50 Hz MFs at 30 *μ*T. Cytosolic superoxide level in C6 cells was not affected by a vertical AC MF but was increased by a horizontal MF (Figure 4). Consistently with previous findings [8], this increase was observed when cytosolic superoxide level was measured 3 h after the end of MF exposure (Figure 4(a)). The effect was observable also in assays performed immediately after MF exposure, but the effect size was somewhat smaller (Figure 4(b)). Viability of the cells (assayed by propidium iodide as described previously [10]) was not affected and was between 96.7% and 98.4% in all groups (data not shown). Mitochondrial superoxide level was not significantly affected by MF exposure in the present experiments (data not shown) and could therefore not be used for comparing the effects of vertical and horizontal MFs.

## 4. Discussion

The most obvious interpretation of the HUVEC results is that a horizontal AC field (nearly perpendicular to the static MF) abolishes the growth-stimulating effect of the static MF. The effect of cancelling the static MF was not tested in the experiments with C6 cells. However, it seems likely that the static geomagnetic field was biologically active also in these experiments, as the effect of the ELF MF was influenced by the relative direction of the static MF. Detection of the geomagnetic field for orientation and navigation is an established effect of static MF. However, animals are believed to use specialized organs for sensing the geomagnetic field, and less is known about detection of MFs in, for example, epithelial and glial cells such as those used in the present study. But putative cell-level detectors such as cryptochromes have been found also in peripheral tissues and in cultured cells [12], so sensitivity to weak MFs could be an intrinsic property of living cells, which has served as the basis for the evolution of a magnetic sense in certain species and in specific organs. Oscillating MFs have been reported to disrupt orientation of both ruminants and birds, and in both cases the effect of the alternating MF seems to depend on its direction in relation to the static MF [4, 13]. Because of the different experimental models, the results cannot be directly compared to those of the present study, and the bird experiments were also done using alternating MFs in the MHz range, where resonances characteristic of the radical pair mechanism are expected [13]. Also biological responses of cultured HUVEC cells, including changes in cellular superoxide levels, were influenced by the relative orientations of a 50 *μ*T static MF and oscillating MFs at the Zeeman resonance of 1.4 MHz [14]. Resonances at the ELF range have been proposed to affect radical pair reactions [15], but further theoretical work is required to understand the in vitro findings of the present study.

Oscillation of the axis of the total MF vector (resultant of the static and ELF fields) has been discussed as a basis for the disruption of magnetoreception by ELF MFs [5]. Our results do not fit with importance of directional oscillation of the MF axis. As the angle between static and vertical ELF fields was 60° in the experiments with HUVEC cells, there was considerable oscillation of the total MF vector also when the ELF field was vertical. In fact, when the static field was 60 *μ*T, the angle of oscillation resulting from the vertical ELF MF (55° variation in direction over a period or the ELF field) was larger than the angle of oscillation (35°) resulting from the horizontal ELF field in combination with the 120 *μ*T static field. What was always higher in the horizontal ELF MF cases was magnitude of the ELF field component that oscillated perpendicular to the static field. In the HUVEC experiments, the perpendicular component was 26 *μ*T (sin⁡60° × 30 *μ*T) when the ELF field was horizontal and 15 *μ*T when the ELF field was vertical. In the C6 experiments, the perpendicular component was 30 *μ*T when the ELF field was horizontal and 5 *μ*T when it was vertical.

It was somewhat surprising that the horizontal ELF MF was found to decrease proliferation even more than the near-zero MF. This finding might be related to incomplete shielding of the geomagnetic field by the *μ*-metal cylinder (stimulation of proliferation by the weak residual MF). Another possible interpretation is that the effect of the horizontal ELF field is not entirely explained by inhibition of the growth-stimulating effect of the static MF. Definite conclusions are not possible without further studies.

In the discussion above, we have assumed that direction in relation to the static magnetic field determines biological effects of ELF MFs. It should be noted that direction in relation to gravity was another difference between the horizontal and vertical ELF MFs. Although there is no mechanism-based reason to assume dependence on gravity, this possibility cannot be excluded and could be investigated in further experiments, for example, by using a horizontal static MF.

The experiments with HUVEC were done using 18 Hz alternating MFs. This frequency was originally chosen because of the suggested resonance mechanisms [13, 16]. To test the validity of the results for power frequency MFs, additional experiments with 50 Hz MFs were performed using C6 glioma cells. This experimental model was chosen based on previous studies, in which we have reported effects of 50 Hz MFs on DNA damage responses, genomic instability, reactive oxygen species, and cell cycle arrest in human and rodent cell lines [8, 10, 17–20]. Horizontally oscillating MFs were used in all these studies, but dependence of the effects on MF direction was not tested. The results of the present study suggest that direction of the ELF field is an important experimental parameter and might be critical for induction of biological effects. It may be of interest that horizontal 50 Hz MFs were used in the recent animal carcinogenicity study reporting positive findings [21]. The results of the present study also show that the difference in the biological effects of vertical and horizontal ELF magnetic fields is similar in two different in vitro experimental models, exposed using two different exposure systems to two different ELF frequencies. It should be noted that the flux density of the ELF fields used in this study did not exceed the flux density of the static field. Direction of the ELF field may not be similarly important in experiments using ELF fields clearly stronger than the ambient geomagnetic field. Further experiments are needed to study the dependence of ELF MF effects on the relative directions and intensities of static and alternating fields.

Of the two proposed mechanisms of magnetoreception, our results are more easily explained by the radical pair mechanism than the magnetite particle hypothesis. Magnetite has been discussed primarily as a basis for animal reception of the direction or inclination of the magnetic field [3], and it is difficult to see how magnetite particles could explain the proliferation-stimulating effect of a 60 *μ*T static MF. Radical pair reactions, in contrast, are known to be sensitive to the intensity of MFs as weak as the geomagnetic field [22], and effects on radical pair reactions in the suggested magnetoreceptor molecules cryptochromes [2] could plausibly affect proliferation through their role in regulation of the cell cycle [12, 23]. However, the radical pair mechanism in its current form sees ELF frequencies as essentially static and does not explain specific effects for ELF fields. In the present study, suppressed proliferation and increased superoxide production were associated with ELF oscillations that were perpendicular to the static MF. This might offer a cue for further theoretical development, as the radical-based magnetoreception mechanism must be direction-sensitive in order to offer a basis for a magnetic compass sense in animals [22]. Much of the research on the radical pair mechanism has focused on cryptochromes as candidate magnetosensor molecules, and MFs are generally assumed to affect light-induced reactions in cryptochromes [1, 22]. However, the experiments of the present and previous studies [6–8, 10, 11, 17–20] were conducted in darkness (in cell culture incubators), indicating a light-independent mechanism. In a recent study specifically designed to test the cryptochrome hypothesis, presence of blue light was not necessary for the biological effects of a horizontal 50 Hz MF, and blue light was actually found to inhibit MF-induced increase of cytosolic superoxide level [24]. It thus appears that MF detection by cultured cells does not require presence of light.

## 5. Conclusions

The results of the present study indicate that the biological effects of a 60 *μ*T static MF can be disrupted by a weaker ELF MF and that the relative direction of the ELF field is critical in this effect. The results also suggest that the difference between the effects of vertical and horizontal ELF magnetic fields is robust: the difference was confirmed in experiments with two different experimental models, using two different exposure systems and both 18 Hz and 50 Hz MFs.

## References

[1] IARC (2002). *Non-Ionizing Radiation, Part 1: Static and Extremely Low Frequency (ELF) Electric and Magnetic Fields*.

[2] Rodgers C. T., Hore P. J. (2009). Chemical magnetoreception in birds: the radical pair mechanism. *Proceedings of the National Academy of Sciences of the United States of America*.

[3] Kirschvink J. L., Walker M. M., Diebel C. E. (2001). Magnetite-based magnetoreception. *Current Opinion in Neurobiology*.

[4] Burda H., Begall S., Červený J., Neef J., Němec P. (2009). Extremely low-frequency electromagnetic fields disrupt magnetic alignment of ruminants. *Proceedings of the National Academy of Sciences of the United States of America*.

[5] Vanderstraeten J., Gillis P. (2010). Theoretical evaluation of magnetoreception of power-frequency fields. *Bioelectromagnetics*.

[6] Martino C. F., Castello P. R. (2011). Modulation of hydrogen peroxide production in cellular systems by low level magnetic fields. *PLoS ONE*.

[7] Martino C. F., Portelli L., McCabe K., Hernandez M., Barnes F. (2010). Reduction of the earth's magnetic field inhibits growth rates of model cancer cell lines. *Bioelectromagnetics*.

[8] Kesari K. K., Juutilainen J., Luukkonen J., Naarala J. (2016). Induction of micronuclei and superoxide production in neuroblastoma and glioma cell lines exposed to weak 50 Hz magnetic fields. *Journal of the Royal Society Interface*.

[9] Pfrommer C. A., Erl W., Weber P. C. (2006). Docosahexaenoic acid induces ciap1 mRNA and protects human endothelial cells from stress-induced apoptosis. *American Journal of Physiology—Heart and Circulatory Physiology*.

[10] Luukkonen J., Liimatainen A., Juutilainen J., Naarala J. (2014). Induction of genomic instability, oxidative processes, and mitochondrial activity by 50Hz magnetic fields in human SH-SY5Y neuroblastoma cells. *Mutation Research—Fundamental and Molecular Mechanisms of Mutagenesis*.

[11] Martino C. F., Perea H., Hopfner U., Ferguson V. L., Wintermantel E. (2010). Effects of weak static magnetic fields on endothelial cells. *Bioelectromagnetics*.

[12] Reddy A. B., Wong G. K. Y., O'Neill J., Maywood E. S., Hastings M. H. (2005). Circadian clocks: neural and peripheral pacemakers that impact upon the cell division cycle. *Mutation Research/Fundamental and Molecular Mechanisms of Mutagenesis*.

[13] Ritz T., Thalau P., Phillips J. B., Wiltschko R., Wiltschko W. (2004). Resonance effects indicate a radical-pair mechanism for avian magnetic compass. *Nature*.

[14] Usselman R. J., Chavarriaga C., Castello P. R. (2016). The quantum biology of reactive oxygen species partitioning impacts cellular bioenergetics. *Scientific Reports*.

[15] Barnes F. S., Greenebaum B. (2015). The effects of weak magnetic fields on radical pairs. *Bioelectromagnetics*.

[16] Zhadin M., Barnes F. (2005). Frequency and amplitude windows in the combined action of DC and low frequency AC magnetic fields on ion thermal motion in a macromolecule: theoretical analysis. *Bioelectromagnetics*.

[17] Kesari K. K., Luukkonen J., Juutilainen J., Naarala J. (2015). Genomic instability induced by 50 Hz magnetic fields is a dynamically evolving process not blocked by antioxidant treatment. *Mutation Research/Genetic Toxicology and Environmental Mutagenesis*.

[18] Luukkonen J., Höytö A., Sokka M. (2017). Modification of p21 level and cell cycle distribution by 50 Hz magnetic fields in human SH-SY5Y neuroblastoma cells. *International Journal of Radiation Biology*.

[19] Luukkonen J., Liimatainen A., Höytö A., Juutilainen J., Naarala J. (2011). Pre-exposure to 50 HZ magnetic fields modifies menadione-induced genotoxic effects in human SH-SY5Y neuroblastoma cells. *PLoS ONE*.

[20] Markkanen A., Juutilainen J., Naarala J. (2008). Pre-exposure to 50 Hz magnetic fields modifies menadione-induced DNA damage response in murine L929 cells. *International Journal of Radiation Biology*.

[21] Soffritti M., Tibaldi E., Padovani M. (2016). Life-span exposure to sinusoidal-50 Hz magnetic field and acute low-dose *γ* radiation induce carcinogenic effects in Sprague-Dawley rats. *International Journal of Radiation Biology*.

[22] Maeda K., Henbest K. B., Cintolesi F. (2008). Chemical compass model of avian magnetoreception. *Nature*.

[23] Kondratov R. V., Antoch M. P. (2007). Circadian proteins in the regulation of cell cycle and genotoxic stress responses. *Trends in Cell Biology*.

[24] Höytö A., Herrala M., Luukkonen J., Juutilainen J., Naarala J. (2017). Cellular detection of 50 Hz magnetic fields and weak blue light: effects on superoxide levels and genotoxicity. *International Journal of Radiation Biology*.

